# Cyst of rete ovarii: A case report

**DOI:** 10.1002/ccr3.6623

**Published:** 2022-11-20

**Authors:** Kritika Jha, Roshan Aryal, Bishal Khaniya, Suvana Maskey, Neebha Ojha

**Affiliations:** ^1^ Department of Obstetrics and Gynecology Tribhuvan University Teaching Hospital Kathmandu Nepal; ^2^ Maharajgunj Medical Campus Institute of Medicine Maharajgunj Nepal

**Keywords:** adnexal mass, ovarian cyst, rete ovarii

## Abstract

Rete ovarii giving rise to cysts, and benign and malignant tumors is a rare entity. Radiological and histopathological examinations can be used to make the diagnosis of rete cyst and differentiate it from cystic tumor of ovarian origin, with surgery being the treatment of choice.

## Graphical abstract

Cyst of rete ovarii is a rare phenomenon which has to be distinguished from cystic ovarian tumor. The treatment of choice is surgery for these cysts.

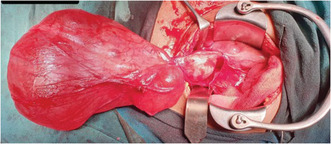

## 1 INTRODUCTION

Rete ovarii (RO), the ovarian congener of the rete testis (RT), develops from cells of mesonephric origin relocating into developing gonads of embryo and is present in the hilus of all ovaries.^1^, ^2^ It is contrived of a grid of uneven clefts, tubules, cysts, and intraluminal papillae lined by flat, cuboidal or columnar epithelium and infrequently ciliated.^1^, ^3^ Tumors deriving from the rete ovarii are an exceedingly sparse origin of the clinically symptomatic adnexal lesions.^1^, ^4^

Here, we report a rare case of cyst of rete ovarii in a 28‐year‐old woman.

## 2 CASE REPORT

A 28‐year‐old unmarried P0 regularly menstruating woman presented with chief complaints of upper abdominal pain, abdominal bloating, and early satiety for 5–6 months. The abdominal pain had no relation to the food intake. There was no history of heartburn, acidic reflux to mouth, fever, jaundice, vomiting, lower limbs swelling, and weight loss. There was no change in the menstrual cycle. There was no family/personal history of malignancy. Past medical history was unremarkable.

On abdominal palpation around 16 weeks size, cystic, non‐tender mass was felt over suprapubic region extending to bilateral iliac fossa with smooth surface, regular margin, freely mobile, could get below the mass and no associated other organomegaly. Per‐vaginal examination was not done. Examination of all other systems was unremarkable.

**FIGURE 1:**
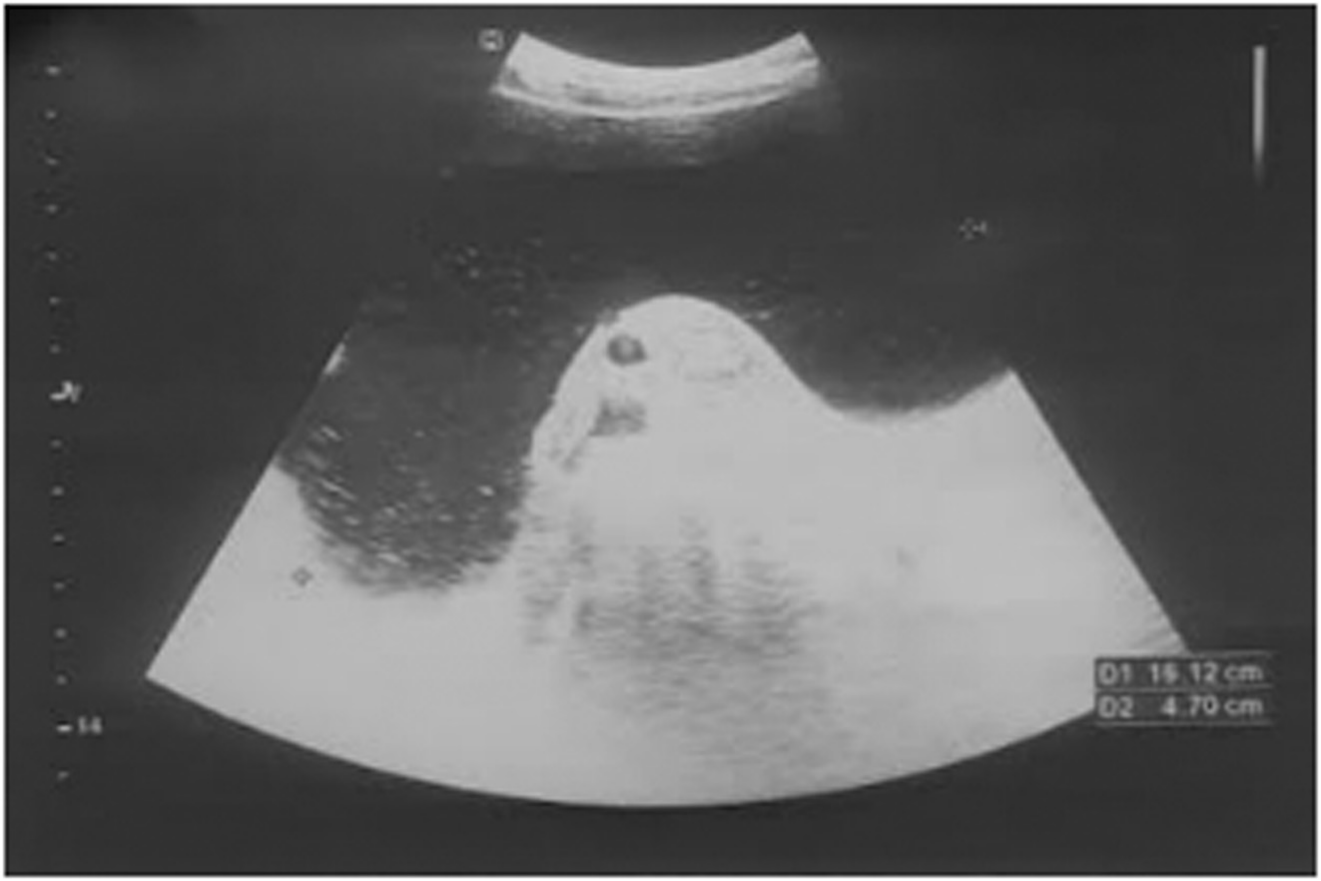
Ultrasonography of abdomen and pelvis shows cystic mass with floating echogenic debris, and no solid component and papillary projection

**FIGURE 2:**
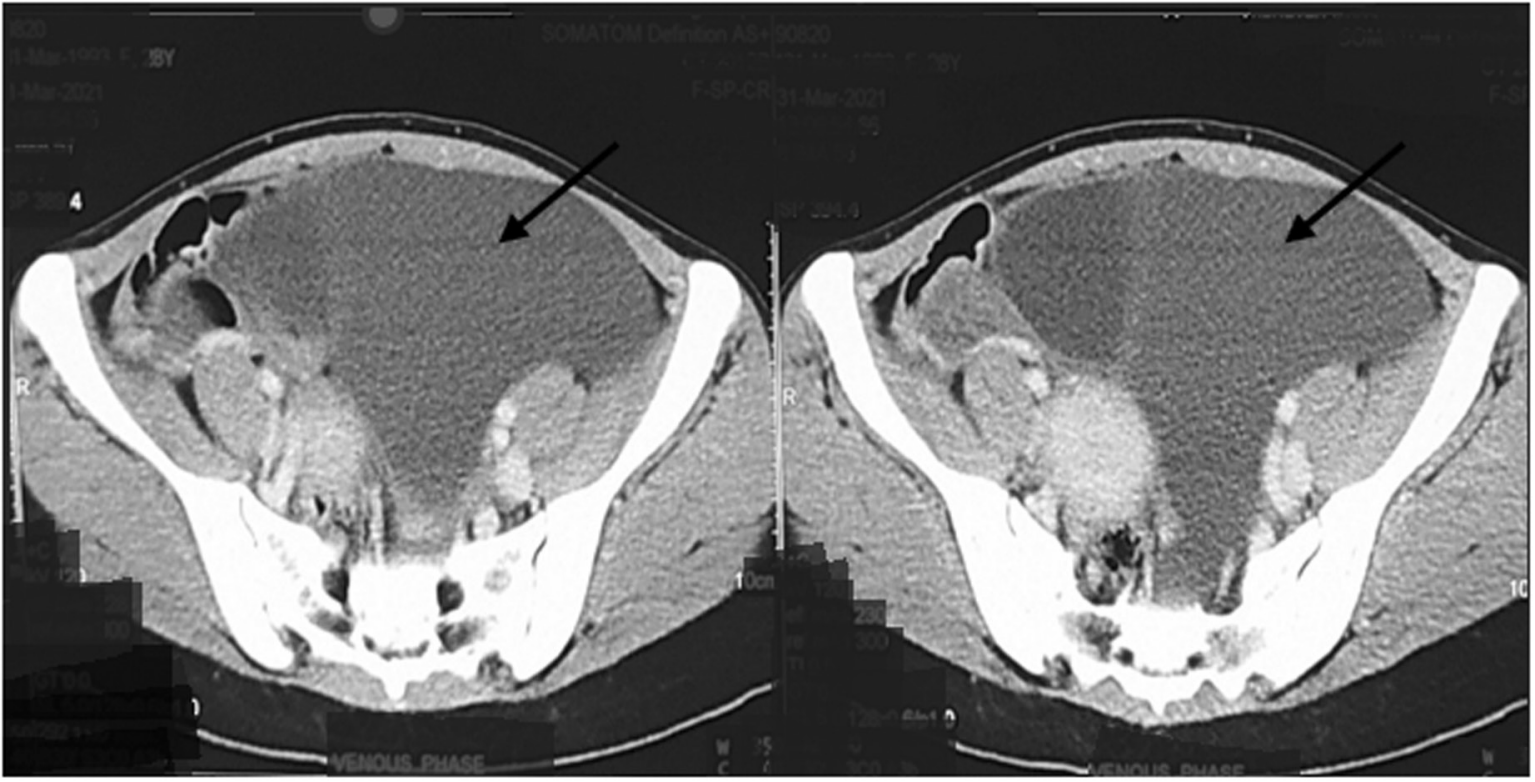
Axial image of contrast‐enhanced computed tomography (CECT) of abdomen/pelvis shows non‐enhancing abdomino‐pelvic cystic mass with no solid component (black arrows)

Ultrasonography of abdomen and pelvis showed thin‐walled abdomino‐pelvic cystic mass of 16.1 cm × 4.7 cm from left adnexa with floating echogenic debris, and no solid component and papillary projection as shown in Figure 1. Blood investigation for the tumor markers showed lactate dehydrogenase (LDH): 226 U/L (normal: 140–280 U/L), beta‐human chorionic gonadotropin (β‐hCG): <2.39 mIU/ml (normal for non‐pregnant: <5.0 mIU/ml), alpha‐fetoprotein (AFP): 1.21 ng/ml (normal: <7.51 ng/ml), carcinoembryonic antigen (CEA): 0.65 ng/ml (normal: <3.0 ng/ml), and Cancer Antigen‐ 125 (CA‐125): 10.8 U/ml (normal: <35.0 U/ml). A contrast‐enhanced computed tomography (CECT) scan of the abdomen/pelvis showed a non‐enhancing abdomino‐pelvic cystic mass measuring 22 cm × 14.7 cm × 15 cm in left side with no solid component, fatty area, or hemorrhagic component, suggestive of peritoneal inclusion cyst or serous cystadenoma as shown in Figure 2.

**FIGURE 3:**
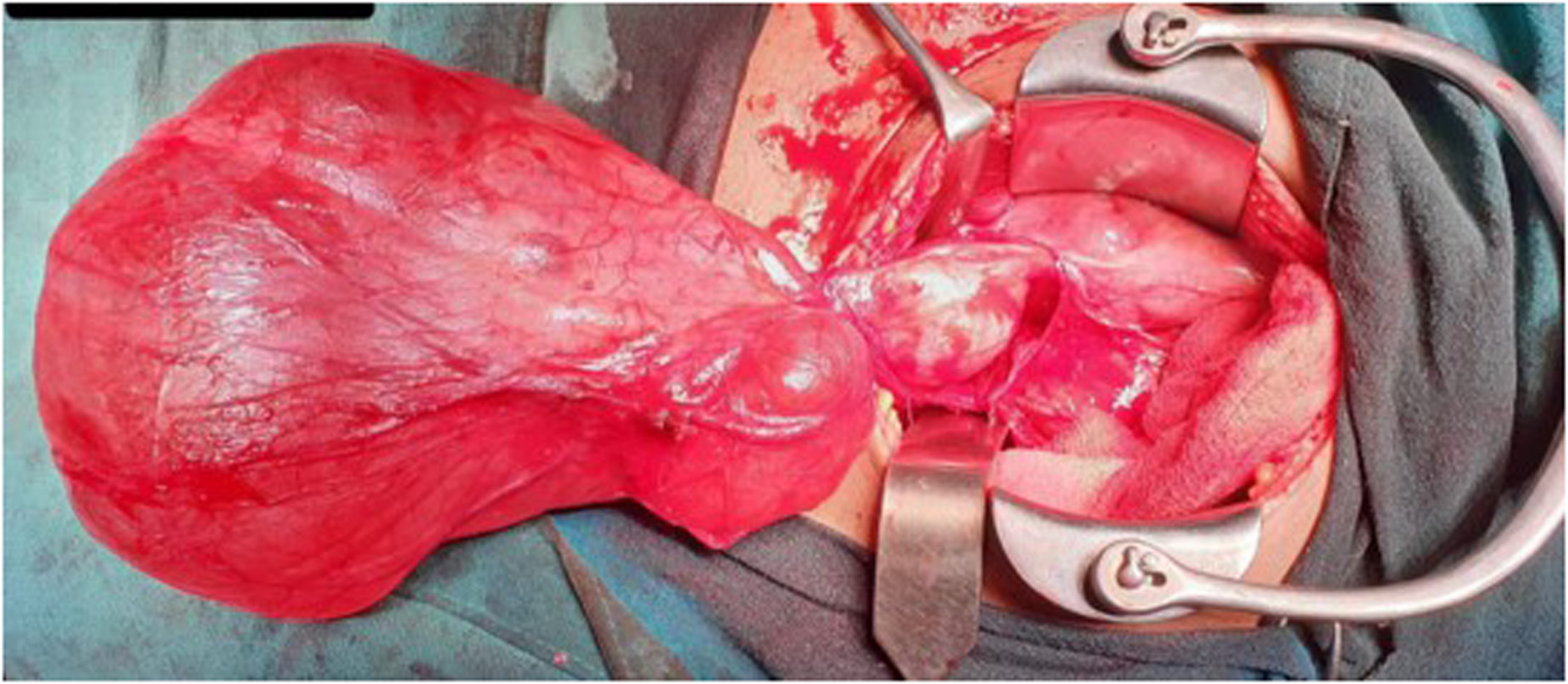
Pre‐operative gross morphological appearance of cystic mass arising from left ovary

**FIGURE 4:**
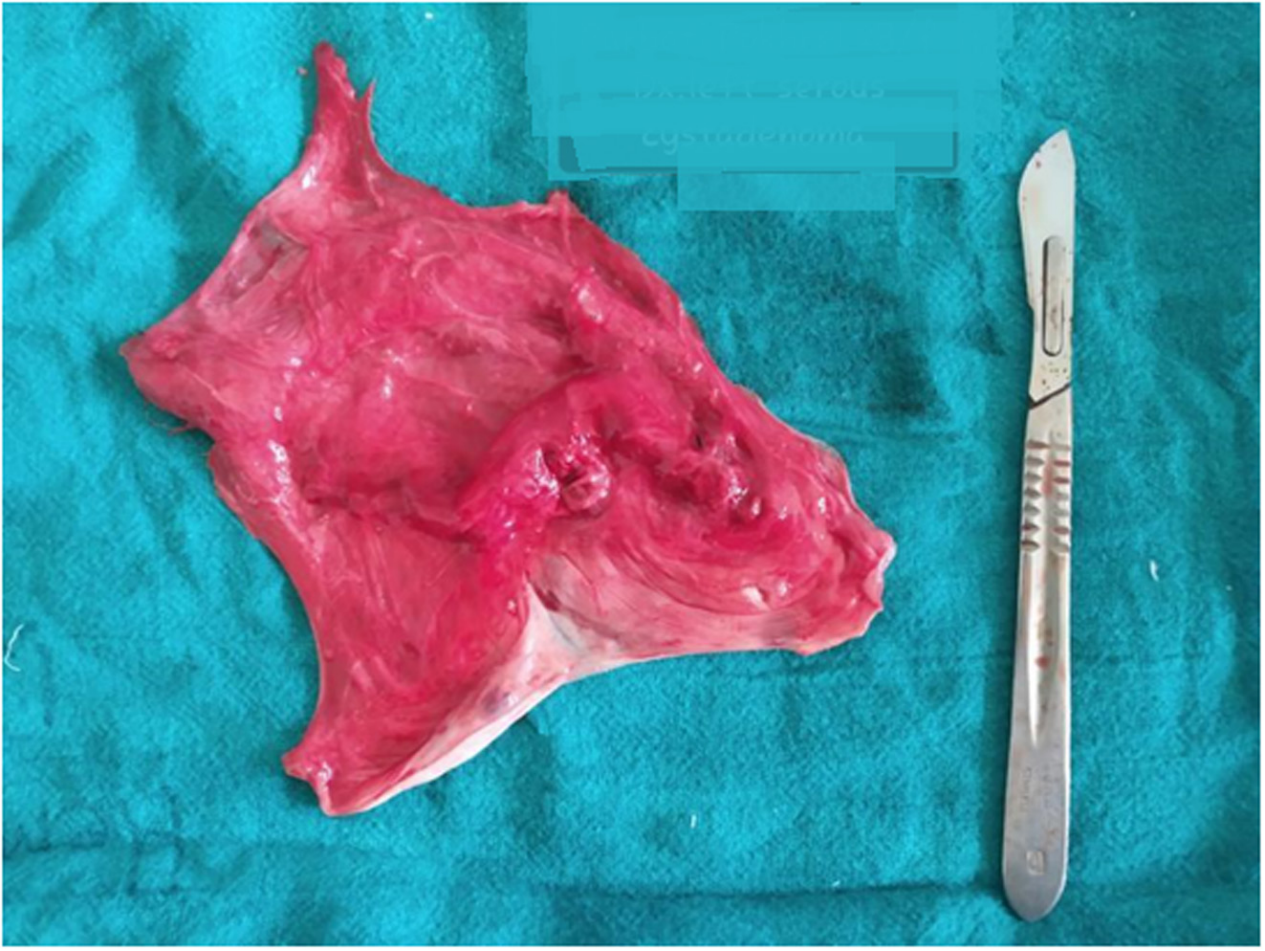
Gross photograph of cyst after cut section showing no surface deposits, solid component, and papillary projection

**FIGURE 5:**
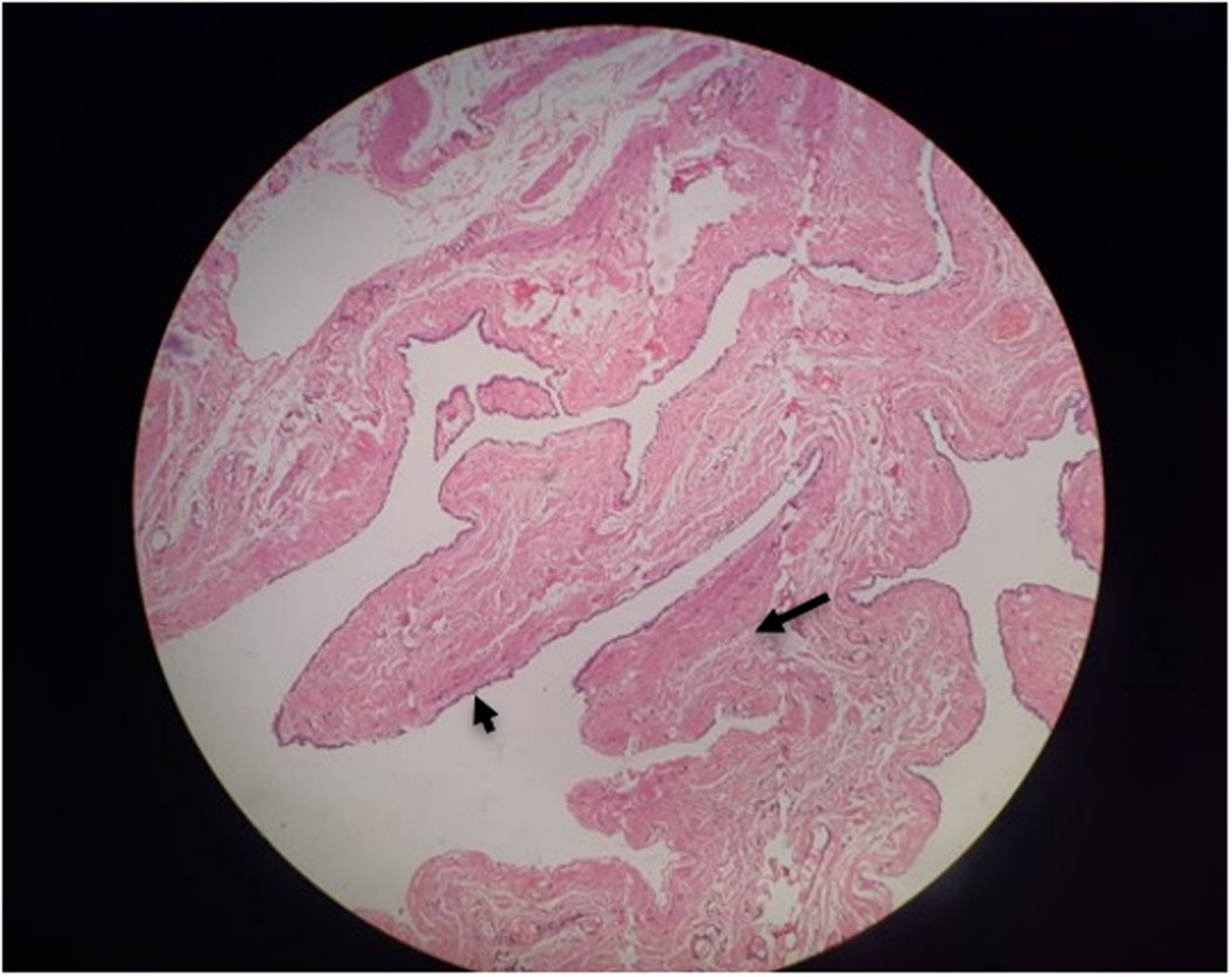
Hematoxylin and eosin (H&E) of a cut section of specimen shows cyst wall lined by flattened to cuboidal epithelium (short black arrow) with subepithelium showing smooth muscle and fibrocollagenous tissue (long black arrow) with no nuclear stratification and atypia

The patient underwent laparotomy and left salpingo‐oophorectomy with a preoperative diagnosis of left serous cystadenoma. Pre‐operatively, there was a single cystic mass arising from the left ovary and twisted once on its pedicle as shown in Figure 3. Left fallopian tube was found to stretch over the mass, with flimsy adhesion present between parietal peritoneum, bowel loops, and the mass for which adhesiolysis was also done. Right fallopian tube, right ovary, and uterus were normal looking. Gross examination of the operative specimen showed 26 cm × 18 cm cyst which on the cut section released serous colored fluid. The capsule of the cyst was intact, and the wall was thin with smooth internal lining and without any solid component and papillary projections as shown in Figure 4. Histological examination showed a cyst wall lined by flattened to cuboidal epithelium with subepithelium showing smooth muscle and fibrocollagenous tissue with no nuclear stratification and atypia as shown in Figure 5. Immunohistochemical study was not done due to unavailability in the in‐patient setting. The lesion was diagnosed as a rete cyst of ovary pathologically.

The postoperative period was unvaried and was discharged on the third postoperative day. During follow‐up examinations, she is doing well and no cyst formation in the contralateral ovary is observed in the 12‐month period after surgery.

## 3 DISCUSSION

Many researchers contemplate RO to be of mesonephric origin, while residue of primordial sex cords in the basal portion of the ovary giving rise to the rete has also been expounded.^1^, ^5^, ^6^, ^7^, ^8^ RO constituting a structure intermediary between the ovarian sex cords and mesonephric duct is also suggested.^5^ RO and RT are anatomical structures of uncertain histogenesis, was stated by Nogales et al., among all this altercation.^4^

Cysts and tumors of RO proper have been reported only rarely. The relative rarity of benign and malignant tumors arising from RO as contrast to other sites in the female genital tract (FGT) has been attributed to reduced levels of estrogen receptor and progesterone receptor in the rete leading to lesser degree of receptiveness to hormone stimulation in this structure and consequently low proliferation rate in the RO.^9^ With the use of antibody to Ki‐67 protein, this reduced proliferation rate was detected, indicating diminished growth rate in RO than in other tissues of the FGT, and this truncated growing potential has been advanced as a theory for uncommonness of tumors in RO.^9^

The appreciably comprehensive details about these cysts were presented by Rutgers and Scully, describing 16 cases of rete cysts in patients ranging from 23 to 80 years (mean age: 59 years), and the cysts ranged from 1 to 24 cm (mean diameter: 8.7 cm).^1^ Majority of the cysts in RO are unilocular and diverge from other ovarian cysts from their hilar location and histopathological finding of miniature crevice‐like outpouchings along the inner surface and a wall often containing clumps of smooth muscle and hyperplastic hilus cells.^10^, ^11^ Rete cyst formation is more common in older than younger women; however, in our case, the age of the patient was 28 years with a unilocular cyst and the largest dimension of the cyst was 26 cm. The relation of cyst to the hilum was veiled, and the presence of distinctive histopathological features of rete cysts eased the diagnosis.

As we noticed in our case, rete cysts can grow to large sizes. This can sometimes increase the risk of torsion. Large cyst can also lead to atrophy of the ovary. The patient ignoring the early symptoms and signs led to development of remarkably large size of cyst in our case. These cysts can be misinterpreted as a cystic tumor of ovarian origin; hence, findings from clinical, radiological, histopathological, and immunohistochemical (although not done in our case) assessment should be integrated to find the exact origin.

Recognition of RO and its lesions is foremost as they can lead to dubiety with other conditions like endometriosis.^6^ A huge help can be gained during arduous differential diagnosis between benign and malignant ovarian lesion if a thorough elucidation and delineation of RO is done.^12^ As they are lined by a single layer of drab epithelium and seem to be amplifications of microscopic rete dilatations, most of the rete cysts are viewed to represent non‐neoplastic dilatations.^1^ In order to avoid needless and undesirable gruesome therapies, identification of benign lesions is cardinal.^13^

## 4 CONCLUSION

Cyst of RO can pose a diagnostic challenge owing to its extreme rarity. The cysts of RO can outreach to large sizes that can lead to various complications, which can be prevented by early accurate diagnosis and treatment.

## AUTHOR CONTRIBUTIONS

Kritika Jha (KJ), Bishal Khaniya (BK), Suvana Maskey (SM), and Neebha Ojha (NO) involved in study concept and provided counseling and surgical therapy to the patient. Kritika Jha (KJ) and Roshan Aryal (RA) collected all the required case information, images, slides, and reports, reviewed the literature, and contributed in both writing and editing the manuscript. BK, SM, and NO served as a senior author and manuscript reviewer. All the authors read and approved the manuscript.

## CONFLICT OF INTEREST

None to declare.

## ETHICAL APPROVAL

Not required.

## CONSENT

Written informed consent was obtained from the patient for publication of this case report and accompanying images. A copy of the written consent is available for review by the editor in chief of this journal on request.

## Data Availability

All the necessary data and materials are within the manuscript.
